# 1-Aminocyclopropane 1-Carboxylic Acid and Its Emerging Role as an Ethylene-Independent Growth Regulator

**DOI:** 10.3389/fpls.2019.01602

**Published:** 2019-12-05

**Authors:** Joanna K. Polko, Joseph J. Kieber

**Affiliations:** Department of Biology, University of North Carolina, Chapel Hill, NC, United States

**Keywords:** 1-aminocyclopropane 1-carboxylic acid, plant physiology, plant hormones, plant signaling, ethylene

## Abstract

1-Aminocyclopropane 1-carboxylic acid (ACC) is the direct precursor of the plant hormone ethylene. ACC is synthesized from *S*-adenosyl-L-methionine (SAM) by ACC synthases (ACSs) and subsequently oxidized to ethylene by ACC oxidases (ACOs). Exogenous ACC application has been used as a proxy for ethylene in numerous studies as it is readily converted by nearly all plant tissues to ethylene. However, in recent years, a growing body of evidence suggests that ACC plays a signaling role independent of the biosynthesis. In this review, we briefly summarize our current knowledge of ACC as an ethylene precursor, and present new findings with regards to the post-translational modifications of ACS proteins and to ACC transport. We also summarize the role of ACC in regulating plant development, and its involvement in cell wall signaling, guard mother cell division, and pathogen virulence.

## 1-Aminocyclopropane 1-Carboxylic Acid as a Precursor of Ethylene

**Figure 1:**
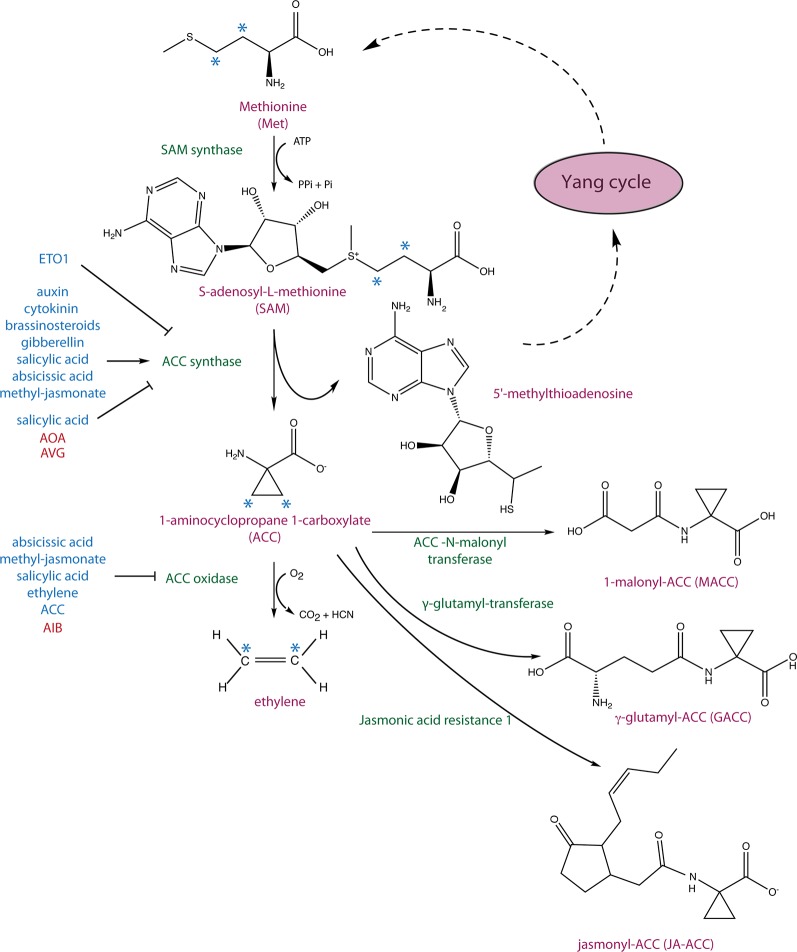
Ethylene biosynthetic pathway and 1-aminocyclopropane 1-carboxylic acid (ACC) conjugation. *S*-adenosyl-L-methionine (SAM) synthase converts methionine to SAM, which is subsequently converted to ACC and 5’-methylthioadenosine (MTA) by ACC synthase (ACS). MTA is recycled back to the Yang cycle to recover methionine, and ACC is oxidized to ethylene by ACC oxidase (ACO). The hormonal inputs that regulate *ACS* and *ACO* expression as well as ACS stability are depicted in blue. ACC has been shown to be converted to three derivates: 1-malonyl-ACC (MACC) by the ACC-N-malonyl transferase, γ-glutamyl-ACC by a glutamyl-transferase, and jasmonyl-ACC (JA-ACC) by jasmonic acid resistance1 (JAR1). The asterisks mark carbons that give rise to ethylene.

Four decades ago, 1-aminocyclopropane 1-carboxylic acid (ACC), a non-proteinogenic amino acid, was discovered to be an intermediate in the biosynthesis of the plant hormone ethylene (Adams and Yang, 1979). Ethylene regulates a wide range of developmental processes and responses to biotic and abiotic stresses, in part by complex interactions with other phytohormones (Muday et al., 2012; Vandenbussche et al., 2012; Merchante et al., 2013; Dubois et al., 2018). Its biosynthesis starts with the conversion of the amino acid methionine to *S*-adenosyl L-methionine (SAM) by SAM synthetase and the subsequent conversion of SAM to ACC, which is catalyzed by ACC synthase (ACS) (**Figure 1**) (Adams and Yang, 1977; Adams and Yang, 1979). The by-product of this reaction, 5’-methylthioadenosine (MTA), is recycled back into the Yang cycle while ACC is oxidized to ethylene by ACC oxidase (ACO) (Murr and Yang, 1975). In *Arabidopsis*, ACO proteins are encoded by five genes (*ACO1–5)*, which belong to a superfamily of oxygenases/oxidases (Dong et al., 1992; Zhang et al., 2004). In general, ACS is the rate-limiting step in ethylene biosynthesis, though in some instances, ACO activity is limiting (Vriezen et al., 1999; Van de Poel et al., 2012). This topic, along with current knowledge on ACO phylogeny and their regulation and importance in agriculture, has been comprehensively discussed in a recent review (Houben and Van de Poel, 2019).

Conjugation of ACC has long been hypothesized to play a role in regulating the level of ethylene biosynthesis but may also generate novel signaling molecules. ACC can be conjugated to 1-malonyl-ACC (MACC), γ-glutamyl-ACC (GACC), jasmonyl-ACC (JA-ACC) (Amrhein et al., 1981; Martin et al., 1995; Staswick and Tiryaki, 2004) (**Figure 1**), and perhaps other yet-to-be discovered conjugates. ACC conjugation regulates the availability of ACC to be converted to ethylene and, therefore, can be utilized by plants to control the level of ethylene biosynthesis. The ACC-to-MACC conversion is catalyzed by the enzyme ACC *N*-malonyl transferase (Martin et al., 1995; Peiser and Yang, 1998). MACC is the most abundant ACC conjugate in ripening *Lycopersicon esculentum* (tomato) fruit, and its formation can be induced by ethylene (Liu et al., 1985; Martin et al., 1995; Peiser and Yang, 1998). ACC was shown to be hydrolyzed back to ACC in *Nasturtium officinale* (watercress) stems, *Nicotiana tabacum* (tobacco) leaf discs, and senescing *Dianthus caryophyllus* (carnation) petals (Jiao et al., 1986; Hanley et al., 1989; ). The formation of GACC is catalyzed by the enzyme γ-glutamyl-transferase (GGT) (Martin et al., 1995; Martin and Slovin, 2000) which, in *Arabidopsis*, is encoded by four widely expressed *GGT* (*1–4*) genes, two of which (*GGT3* and *GGT4)* encode catalytically inactive or minimally active enzymes. Interestingly, GGT1 and GGT2 appear to be localized extracellularly (Martin et al., 2007).

JA-ACC is the second most abundant JA conjugate detected in *Arabidopsis* leaves and is formed by JAR1, a JA-amino synthetase. Similar to MACC and GACC, JA-ACC might regulate levels of ACC available for the biosynthesis of ethylene, and may also regulate JA levels in the plant (Staswick and Tiryaki, 2004). The availability of ACC can also be controlled by plant and bacterial encoded ACC deaminases that irreversibly convert ACC to ammonia and α-ketobutyric acid (Glick et al., 1998). Multiple species of plant growth–promoting bacteria from various phyla, including *Proteobacteria*, *Actinobacteria*, *Firmicutes*, and *Bacteroidetes*, contain ACC deaminases that can decrease host plant ACC levels. A decrease of ACC often facilitates stress-coping mechanisms under various unfavorable conditions (reviewed in Glick, 2014; Nascimento et al., 2014; Van de Poel and Van Der Straeten, 2014). For example, tomato plants grown in the presence of ACC deaminase–producing *Enterobacter* or *Pseudomonas* strains exhibit an increased tolerance to flooding stress, likely as a result of decreased ethylene (Grichko and Glick, 2001a). Moreover, *Arabidopsis*, *Populus* (aspen), and tomato plants have been shown to contain ACC deaminases, but their role in plant growth and development has not as yet been elucidated (McDonnell et al., 2009; Plett et al., 2009).

ACS is generally encoded by a multigene family in most plant species. For example, in *Arabidopsis*, ACS proteins are encoded by a family of 12 genes, though only *ACS2–ACS9* and *ACS11* encode functional ACS enzymes; *ACS10* and *ACS12* encode aminotransferases (Liang et al., 1995; Yamagami et al., 2003), *ACS3* is a pseudogene, and ACS1 is catalytically inactive due to deletion of a highly conserved tripeptide Thr–Asn–Pro (TNP) (Liang et al., 1995). The remaining *Arabidopsis* ACS proteins can potentially form up to 45 different functional homo- and heterodimers, comprising a family of ACS enzymes with diverse biochemical properties (Tsuchisaka and Theologis, 2004; Tsuchisaka et al., 2009). ACS enzymes share an N-terminal catalytic domain and can be divided into three classes based on the presence of regulatory residues within their C-termini (Harpaz-Saad et al., 2012). The C-terminal domain of type-1 ACSs (ACS2 and ACS6 in *Arabidopsis*) have target residues for both calcium-dependent and mitogen-activated protein kinases (CDPKs and MAPKs, respectively) (Liu and Zhang, 2004; Sebastià et al., 2004). Joo et al. (2008) showed that phosphorylation of the Ser residues within the C-terminus of ACS6 by MPK6 increases its stability and is associated with increased rates of ethylene production. Type-2 ACS proteins have a target site for CDPKs and an overlapping Target of ETO1 (TOE) motif. Type-2 ACSs are targeted for degradation by the 26S proteasome pathway upon binding of ETHYLENE OVERPRODUCER1 (ETO1) or one of its paralogs, ETO-like1 or 2 (EOL1/2) (Chae et al., 2003; Wang et al., 2004; Christians et al., 2009; ). Phosphorylation of the C-terminus of type-2 ACS proteins reduces their targeting for degradation. Type-3 ACS proteins (ACS7 in *Arabidopsis*) have a short C-terminus that lacks an apparent regulatory domain, though ACS7 levels can be mediated through the activity of the E3 ligase XBAT32 (Prasad et al., 2010; Lyzenga et al., 2012). The 14-3-3 phospho-specific binding proteins bind various ACS isoforms *in planta* to regulate their stability. 14-3-3s also bind to ETO1 and EOL2, but in these cases, it results in their destabilization. Therefore, 14-3-3s control the level of ACS proteins through a bipartite mechanism—on one hand stabilizing them through direct binding, but also destabilizing the ubiquitin ligases involved in their degradation (Yoon and Kieber, 2013).

There is a complex crosstalk between ethylene and other plant hormones at the level of signaling and/or biosynthesis, the latter of which includes both transcriptional and post-transcriptional regulation of ACS (reviewed in Kazan and Manners, 2012; Muday et al., 2012; Van de Poel et al., 2015; Shigenaga and Argueso, 2016; Hu et al., 2017; Liu et al., 2017; Zemlyanskaya et al., 2018; Bürger and Chory, 2019; Qin et al., 2019). For example, cytokinin and brassinosteroid additively increase the stability of type-2 ACS proteins independently of their TOE domains (Hansen et al., 2009). The effect of various phytohormones on the rates of degradation of type-1, -2, and -3 ACS proteins was comprehensively investigated using etiolated *Arabidopsis* seedlings as a model (Lee et al., 2017). Consistent with previous results, auxin was found to increase *ACS2* and *ACS5* transcript levels (type-1 and type-2, respectively) as well as to stabilize their encoded proteins. Gibberellin, brassinosteroid, and cytokinin were also found to decrease the turnover of ACS2 and ACS5 proteins. Salicylic acid (SA) stabilized ACS5 but decreased the stability of ACS2 protein, the latter of which is different from the effect in light-grown seedlings in which SA stabilizes ACS2 (Liu and Zhang, 2004; Lee et al., 2017). Abscisic acid (ABA) and methyl-jasmonate (MeJA) did not affect ACS2 protein levels but increased the stability of ACS5, which is distinct from the negative effect of these hormones on levels of ethylene production (Lee et al., 2017). The reduced ethylene biosynthesis in response to ABA and MeJA is likely explained by the downregulation of *ACO* genes in response to these hormones (Lee et al., 2017). Interestingly, the turnover of ACS7 (a type-3 ACS) was not regulated by any of the hormones examined in the study, and the analysis of its half-life showed that ACS7 is the most stable protein, confirming previous suggestions (Chae and Kieber, 2005). Moreover, the heterodimerization with ACS7 increased the stability of both ACS2 and ACS5 as compared to the respective homodimers, which suggests that dimerization among various ACS isoforms may regulate their turnover rate and, as a result, ethylene biosynthesis (Lee et al., 2017). ACS5 proteins are also stabilized when etiolated *Arabidopsis* seedlings are moved to the light, promoting ethylene biosynthesis and hypocotyl elongation during this transition (Seo and Yoon, 2019).

## ACC Transport and LYSINE HISTIDINE TRANSPORTERS

Ethylene is involved in various stress-related responses such as wounding, pathogen infection, neighbor proximity, elevated temperatures, drought, soil waterlogging, and submergence (Vandenbussche et al., 2005; Sasidharan and Voesenek, 2015; Huang et al., 2016; Loreti et al., 2016; Valluru et al., 2016; Dubois et al., 2018). Following the demonstration that ethylene leads to epinasty of petioles in waterlogged tomato plants (Jackson and Campbell, 1975), Bradford and Yang showed that waterlogging and root anoxia correlated with the shootward transport of ACC, its subsequent conversion to ethylene, and leaf epinasty (Bradford and Yang, 1980). This spatial separation between the biosynthesis of ACC and the its conversion to ethylene is the result of the oxygen dependence of the ACO enzyme (Murr and Yang, 1975). Multiple studies confirmed the phenomenon of ACC transport between roots and shoots in several plant species (e.g. Else and Jackson, 1998). During the de-submergence of flood-tolerant *Rumex palustris* (marsh dock) plants, ACC delivered from the root contributes to the pool of ACC that accumulated in the shoot during submergence to stimulate petiole elongation (Voesenek et al., 2003). In contrast, flood-intolerant *Rumex acetosa* (common sorrel) does not accumulate ACC in roots or shoots and consequently fails to recover from the detrimental effects of flooding. The root-to-shoot transport of ACC is thought to occur primarily in the xylem, though there is evidence for phloem-translocated ACC as well (Amrhein et al., 1981; Hume and Lovell, 1983). Radio-labeled ACC application to the abaxial side of *Gossypium hirsutum* (cotton) leaves resulted in both basipetal and acropetal transport of ACC throughout the plant as well as rapid conversion to [^14^C]MACC, which was not translocated from the source leaf. ACC is compartmentalized within the tonoplast of *Zea mays* (maize) leaf mesophyll cells *via* a mechanism dependent on an electrochemical gradient (Saftner and Martin, 1993). Translocation of ACC conjugates into the vacuole likely plays a role in regulating ACC availability and/or ethylene levels. In *Acer pseudoplatanus* (sycamore maple) protoplasts treated with [^14^C]ACC, there was a steady transport of the [^14^C]MACC conjugate into the vacuole (Bouzayen et al., 1988). Furthermore, Tophof et al. (1989) showed that MACC accumulated to higher levels than ACC in vacuoles in both wheat (*Triticum aestivum*) and barley (*Hordeum vulgare*) plants.

The ability of plants to transport ACC both within the cell and throughout the plant suggests the existence of ACC transporters. ACC and its structural analog α-aminoisobutyric acid (AIB) are taken up by tomato pericarp cells; this uptake is inhibited by neutral but not by acidic or basic amino acids (Saftner and Baker, 1987). Tophof et al. (1989) speculated that ACC might be translocated to the tonoplast by a neutral amino acid transporter and as MACC competitively inhibited the transport of malate to the vacuole, they suggested that these molecules likely shared a common translocator. Recently, the identification of an ACC-resistant (*are2*) *Arabidopsis* mutant that displayed a reduced uptake of exogenous ACC led to the identification of the LYSINE HISTIDINE TRANSPORTER1 (LHT1) as a potential ACC transporter (Shin et al., 2014). LHT1 localized to the plasma membrane of leaf mesophyll and epidermal root cell and is not detected in the vasculature (Chen and Bush, 1997; Hirner et al., 2006). The *lht1* mutant displays severe growth defects on media with aspartate and glutamate as the sole nitrogen source and is impaired in the uptake of [^14^C]-labeled amino acids. The *are2* mutant, allelic to *lht1-5*, was resistant to ACC but displayed a normal triple response when exposed to ethylene. Isolated *are2/lht1-5* protoplasts display reduced accumulation of [^14^C]ACC. Additionally, competition experiments showed that the presence of alanine and glycine can reduce the triple response morphology elicited in response to ACC, consistent with Tophof’s (1989) speculation that ACC is translocated through the tonoplast by a neutral amino acid transporter. However, Hirner et al. (2006) showed that lysine and histidine are the best substrates for LHT1, suggesting that multiple distinct transporters may act in the movement of ACC. It is possible that the ACC uptake and transport are mechanistically different and require distinct transport proteins. The subject of alternative ACC transporters and strategies to identify them has been recently discussed in a comprehensive review (Vanderstraeten and Van Der Straeten, 2017). Further studies are needed to identify and distinguish transporters involved in the short- and long-distance ACC translocation, its uptake into cells, as well as its intracellular trafficking.

## ACC in Plant Development and Beyond

A growing body of evidence indicates a role for ACC as a signaling molecule distinct from its role in ethylene biosynthesis. One of the first findings consistent with this was the discovery of the involvement of ACC in the regulation of cell wall function in the FEI pathway (Xu et al., 2008). FEI1 and FEI2 are leucine-rich repeat receptor-like kinases (LRR-RLKs) that have been linked to cellulose biosynthesis. *fei1 fei2* loss-of-function mutants display root swelling under high concentrations of salt and sucrose, decreased biosynthesis of cellulose, hypersensitivity to the cellulose inhibitor isoxaben, thickening of etiolated hypocotyls, and a decrease in the formation of cellulose rays in seed coat mucilage (Xu et al., 2008; Harpaz-Saad et al., 2011), which together indicate a role of the FEI proteins in regulating cellulose biosynthesis. Intriguingly, inhibition of ethylene biosynthesis [via aminooxy-acetic acid (AOA) or AIB; **Figure 1**] reverted the swollen root phenotype of *fei1 fei2* mutants, but blocking ethylene perception, using either the inhibitors 1-methylcyclopropane (1-MCP) or silver thiosulfate, or by introducing ethylene-insensitive *ein2* and *etr1* mutations into the *fei1 fei2* background, had no effect. Furthermore, the FEI kinase domain was shown to directly interact with type-2 ACS proteins, suggesting a direct link to ACC synthesis (Xu et al., 2008).

An analysis of cell elongation in roots treated with the cellulose biosynthesis inhibitor isoxaben provided further support that ACC acts as a signal. Tsang et al. (2011) analyzed root trichoblast length upon treatment with isoxaben in presence or absence of various ethylene inhibitors. Interestingly, inhibitors of ethylene biosynthesis [amino-ethoxyvinylglycine (AVG), AOA, or 2-anilino-7-(4-methoxy-phenyl)-7,8-dihydro-5(6*H*)-quinazolinone] reversed the isoxaben-induced elongation defects, but inhibitors of ethylene perception did not. Moreover, short-term ACC treatment induced shortening of trichoblasts in ethylene-insensitive *ein3 eil1* mutants, further supporting the hypothesis that ACC acts independently of ethylene signaling. Additionally, the authors found that both cell wall damage–induced and ACC-mediated growth inhibition is dependent on auxin signaling since the growth inhibition was absent when combined with α-(phenylethyl-2-one)- indole-3-acetic acid (PEO-IAA), a transport inhibitor response 1 (TIR1) receptor antagonist This is consistent with the suppression of the *fei1 fei2* root swelling phenotype by auxin biosynthesis mutants (Steinwand et al, 2014). Together, these studies suggest that ACC plays a role in the response to cell wall perturbations, triggered by either chemical or genetic disruption of cellulose synthesis, and that auxin is involved in this pathway (Xu et al., 2008; Tsang et al., 2011).

Genetic analysis of disruption of *ACS* genes in *Arabidopsis* also supports a function for ACC in addition to its role as an ethylene biosynthetic precursor. A comprehensive genetic study of all members of the ACS gene family in *Arabidopsis*, including the generation and analysis of single, double, triple, and high-order *acs* mutants, suggested novel roles for ACS beyond ethylene biosynthesis (Tsuchisaka et al., 2009). Analysis of the mutants revealed both synergistic and antagonistic relationships among various *ACS* genes in ethylene biosynthesis and in regulation of hypocotyl and rosette growth, and flowering time. Disruption of multiple *ACS* genes led to a progressive increase in plant size, concomitant with a decreased level of ethylene biosynthesis. Remarkably, an octuple *acs 2, 4, 5, 6, 7, 9, amiR acs8 acs11* mutant, which had a ~90% decrease in the level of ethylene production, displayed embryonic/gametophytic lethality and/or unfertilized ovules. The octuple *acs* mutant inflorescences are significantly taller than wild-type or lower-order *acs* mutants, despite their initial reduced growth rate. An independent octuple mutant line analyzed in the study could only be propagated when the amiR transgene was heterozygous, consistent with embryo/gametophytic lethality or infertility. The striking phenotypes of the octuple *acs* mutant is distinct from the full reproductive viability of even very strong ethylene signaling mutants, suggesting that *ACS* genes play a role beyond acting as precursors in ethylene biosynthesis. Alternatively, ACS proteins may have a moonlighting function, and the reported lethality of the octuple mutants may result from disruption of an unrelated process. The precise nature of the embryo/gametophyte lethality of the octuple *acs* mutant needs further characterization.

ACC was recently shown to play a role in stomatal development. The terminal division of the guard mother cell (GMC) produces the two guard cells (GCs) that comprise the mature stomata (Yin et al., 2018). Application of the ACS inhibitor AVG induced the formation of pore-less, single guard cell (SGCs). Similarly, the octuple *acs 2, 4, 5, 6, 7, 9, amiR acs8 acs11* mutant (Tsuchisaka et al., 2009) developed SGCs. This SGC phenotype was not observed in the presence of ACO inhibitors (AIB or Co^2+^) or the ethylene binding inhibitor 1-MCP, nor was it present in various ethylene-signaling mutants (*etr1*, *ein2, ctr1, ein3 eil2*). ACC did not increase cell division in GMCs in wild-type plants but did so (as did ethylene) in *fama* and *four lips* (*flp*)/*myb88* mutants, which developed clusters of thin cells on the epidermis. FAMA and FOUR LIPS (FLP)/MYB88 are central regulators of the last cell division of GMC, acting upstream of the core cell cycle genes (*CYCA2;3*, *CDKB1;1*, and *CDKA;1*) (Xie et al., 2010; Vanneste et al., 2011; Yang et al., 2014). Because ACC induced extra divisions in these mutant backgrounds, it was concluded that FAMA and FLP/MYB88 might antagonize the effect of ACC on the GMC division. Moreover, ACC, but not ethylene, stimulated the expression of *CYCA2;3* and *CDKB1;1* in *fama* and *flp/my88* mutants, and conversely, AVG downregulated the expression of these genes. ACC partially rescued the SGC formation in the *acs 2, 4, 5, 6, 7, 9, amiR acs8 acs11* line, but not in *cyca2;3* and *cdkb1;1* mutants, suggesting that it acts upstream of the cell cycle–dependent control of the GMC division.

Recent studies suggest that the signaling role of ACC could extend beyond the plant kingdom. Since many plant growth–promoting rhizobacteria (PGPR) possess ACC deaminase genes and utilize ACC as a source of nitrogen, Li et al. (2019) examined whether ACC could act as a chemoattractant. Indeed, *Pseudomonas putida* displayed a chemotactic response to ACC, but not to ethylene, and the ability to respond to ACC was correlated to the ability of *P. putida* to colonize wheat roots.

The fungal pathogen, *Verticillium dahliae* is a soil-borne pathogen of many plant species, causing vascular wilt disease. Tsolakidou et al. (2019) found that genetic modulation of ACC levels in *V. dahliae* affected its microsclerotia development and hyphae growth. Overexpression of ACC deaminase in *V. dahliae* led to increased virulence on tomato and eggplant, including enhanced wilting and greater fungal growth. On the contrary, the disruption of ACC deaminase in *V. dahliae* resulted in reduced virulence and less fungal biomass. To test if ACC was acting as a signal controlling plant defense, wild-type and the ethylene-insensitive mutant *Never-ripe* (*Nr*) tomato plants were treated with ACC and then infected with *V. dahliae*. ACC increased the resistance of both wild-type and *Nr* plants, suggesting that ACC and not ethylene promotes disease resistance against *V. dahliae*. It will be interesting to investigate if similar responses occur in other plant–pathogen interactions.

## Conclusions and Outstanding Questions

**Figure 2:**
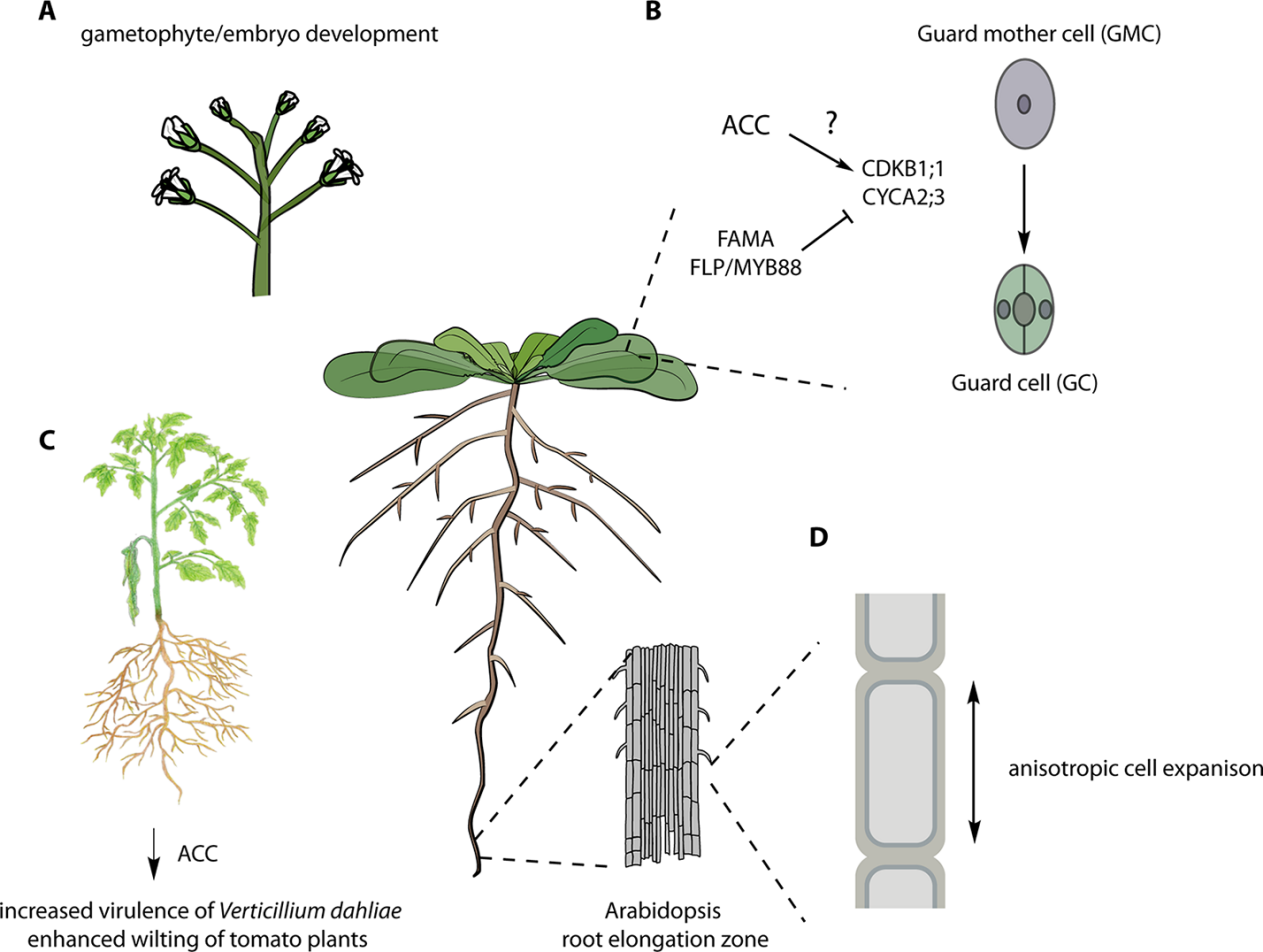
Various ACC-dependent processes. **(A)** The infertility of the octuple *acs 2, 4, 5, 6, 7, 9, amiR acs8 acs11* mutants suggests an essential role in gametophytic and/or embryonic function (Tsuchisaka et al., 2009). **(B)** Role of ACC in stomatal development. ACC is required for the division of the guard mother cell (GMC) likely *via* the regulation of the indicated cell cycle regulators (Shin et al., 2014). **(C)** Overexpression of the ACC deaminase from the fungal pathogen of tomato, *Verticillium dahliae*, enhances its virulence, and pre-treatment of tomato plants with ACC reduced the symptoms of *V. dahliae* infection even in ethylene-insensitive mutants (Tsolakidou et al., 2019). **(D)** ACC is involved in cell wall signaling regulating anisotropic elongation of root cells (Xu et al., 2008; Tsang et al., 2011). See text for more details. Figure adapted, with permission, from Figshare [**A** (Bouché, 2018a); **B** (Bouché, 2018b); **C** (Davis and Mitra, 2019); **D** (Bouché, 2017)].

An increasing number of studies have established that ACC acts as a signaling molecule beyond its function in ethylene biosynthesis (**Figure 2**). ACC appears to be involved in regulating multiple processes, including stress responses, cell expansion, cell wall function, stomatal development, pathogen interactions, and fertilization-related events. Additional studies are needed to elucidate the mode of action of ACC and to further define its role in plant growth and development. For example, the embryo/gametophyte lethality of the octuple *acs* mutants (Tsuchisaka et al., 2009) raises questions about the precise basis for ACC action during gametophyte development, fertilization, or embryogenesis. The recent link between LHT and ACC uptake (Shin et al., 2014) provides tantalizing clues to the mechanisms of ACC uptake/transport, but as LHT is part of a large gene family, other amino acid transporters may also participate in ACC uptake and translocation (reviewed in Vanderstraeten and Van Der Straeten, 2017). The biological roles of ACC derivatives, including the JA-ACC, GACC, and MACC, also need to be investigated. Finally, in light of the findings presented in this review, studies that use ACC as a proxy for ethylene need to be interpreted with caution.

## Author Contributions

Both authors wrote the manuscript.

## Funding

This work has been supported by grant IOS-1856431 from the National Science Foundation.

## Conflict of Interest

The authors declare that the research was conducted in the absence of any commercial or financial relationships that could be construed as a potential conflict of interest.
